# Ergogenic Effects of Green Tea Combined with Isolated Soy Protein on Increasing Muscle Mass and Exercise Performance in Resistance-Trained Mice

**DOI:** 10.3390/nu13124547

**Published:** 2021-12-18

**Authors:** Mon-Chien Lee, Yi-Ju Hsu, Li-Hsuan Yang, Chi-Chang Huang, Chun-Sheng Ho

**Affiliations:** 1 Graduate Institute of Sports Science, National Taiwan Sport University, Taoyuan 33301, Taiwan; 1061304@ntsu.edu.tw (M.-C.L.); ruby780202@ntsu.edu.tw (Y.-J.H.); 1070211@ntsu.edu.tw (L.-H.Y.); 2 Division of Physical Medicine and Rehabilitation, Lo-Hsu Medical Foundation, Inc., Lotung Poh-Ai Hospital, Yilan 26546, Taiwan; 3 Department of Physical Therapy, College of Medical and Health Science, Asia University, Taichung 41354, Taiwan

**Keywords:** isolated soy protein, green tea, muscle mass, resistance training, exercise performance

## Abstract

It is well known that supplementation with high protein after exercise can effectively promote muscle synthesis and repair, while green tea is rich in catechins that have antioxidant effects. We aimed to explore the effects of green tea combined with isolated soy protein on increase muscle mass in resistance-trained mice. A total of 32 male ICR mice (8-weeks old) were divided into four groups (*n* = 8/group), sedentary control group (SC), isolated soy protein with green tea group (ISPG), resistance training group (RT), isolated soy protein and green tea combine with resistance training group (ISPG + RT). All mice received control or ISPG by oral gavage for four consecutive weeks. Forelimb grip and exhaustive swimming time were used for exercise performance evaluation. In biochemical profile, we analyzed lactate, ammonia, blood urea nitrogen (BUN), and glucose and muscle damage index creatine kinase (CK) after exercise as biochemical parameters of exercise fatigue. The grip strength, muscular endurance, and exhaustive swimming time of the ISPG + RT group were significantly increased than other groups (*p* < 0.05), and also significantly decreased in serum lactate and ammonia levels (*p* < 0.05, respectively). The ISP + RT group was not only increased in quadriceps weight, (*p* < 0.05) but also decreased EFP (*p* < 0.05). We recommend using a 4-week supplementation with ISPG, combined with RT, to increase muscle mass, exercise performance, glycogen storage, and reduce fatigue biochemical parameters after exercise. The benefits of long-term supplementation or application to human supplementation can be further explored in the future.

## 1. Introduction

Athletes and many amateur sports enthusiasts like to take some protein products after a training session, to increase their dietary protein intake and compensate protein loss in training. It is generally accepted that essential amino acid (EAA) and isolated protein are important in rates of muscle protein synthesis (MPS) [1]. A recent study showed that a high-protein diet was involved in several benefits of MPS [2]: (1) Greater improvement in muscle mass with regular resistance training. (2) Attenuating muscle loss when minimizing caloric intake. (3) Attenuating age-associated muscle loss. When compared with training alone, post-training supplementation with EAA or high-quality protein (whey protein, soy protein and casein) has a greater effect on muscle maintenance, repairing and building [1].

There are numbers of protein powder products on the market aimed at increasing muscle mass and improving exercise performance. Most people prefer the animal-type product (whey protein) rather than the plant ones, in consideration of the ability of muscle building. In fact, soy protein is a complete protein which contains all the EAAs for humans and has exhibited its remarkable nutritional value and physiological function in human [3]. Isolated soy protein (ISP) is a separated compound of soybean which contain 90% protein and is nearly carbohydrate and fat-free. It is rich in a sufficient amount of branched-chained amino acids (BCAAs) included leucine, isoleucine, and valine, which are also the antioxidants which are compounded that inhibit the oxidation in cells of organisms which are caused by the free radical [4]. In addition, the isoflavones and saponin components contain in the ISP have activities of antioxidant, anti-inflammation, immune regulation, and cardio-protection, which may ameliorate the muscle damage and fatigue caused by the free radical yield in exercise [5]. Therefore, both the amino acids and phytochemicals in isolated soy protein are important to speed up the rate of MPS and recovery from exercise.

Green tea is a well-known beverage made from the plant *Camellia sinensis* leaves. It exhibits several favorable properties in many diseases and health. Catechin is the main functional ingredient in tea, in the form of *epigallocatechin* (EGC), epicatechin gallate (EGG), epigallocatechin gallate (EGCG), *epicatechin* (EC), *catechin gallate* (CG), *gallocatechin gallate* (GCG) and *catechin*. EGCG is the most abundant catechin in green tea, accounting for 50–80% of catechin. Some research shows that EGCG can effectively maintain cell integrity during tumor development by enhancing gap junction communication between cells and exhibits strong activity of anti-radicals to suppress the cell proliferation [6].

In summary, for BCAA-rich ISP, it can effectively increase muscle growth, recovery and MPS, as well as green tea catechins that effectively remove oxidative free radicals after exercise. We hypothesized that 4-weeks supplementation of ISP combined with green tea and conduct resistance training will improve muscle mass and strength and improve endurance performance in mice.

## 2. Materials and Methods

### 2.1. Composition of ISP with Green Tea

The ISP with green tea (ISPG) was obtain from National Taiwan Sport University (Taoyuan, Taiwan), which contained 5% Jin Xuan green tea powder (Taiwan Tea var. 12) from Pinling (New Taipei, Taiwan) and 95% isolated soy protein from Bestjet Biotechnology Co., Ltd. (New Taipei, Taiwan). SGS Taiwan, Ltd. (New Taipei, Taiwan) had analyzed the types of nutrients and catechins present in green tea powder products (Table 1). Similar to previous studies on nutrients and amino acids present in ISP products [7].

### 2.2. Animals and Experimental Design

6-week-old male ICR mice purchased from BioLASCO (Yilan, Taiwan). Animals were housed in the animal facility of Graduate Instituted of Sport Science at National Taiwan Sport University, on condition that room temperature and relative humidity were 23 ± 2 °C and 50~60%, respectively, with a 12-h light–dark cycle. During the experiment, all mice were given distilled water and standard laboratory food (No. 5001; PMI Nutrition International, Brentwood, MO, USA), which has 3.36 kcal (58.00% carbohydrate, 13.50% fat and 28.50% protein. The protein source was from soybean, fish, oats, dried whey, etc.), ad libitum. Animal experimentation and procedure were approved by The Institutional Animal Care and Use Committee (IACUC) of National Taiwan Sport University, Taoyuan City, Taiwan (IACUC No. 10718).

**Figure 1:**
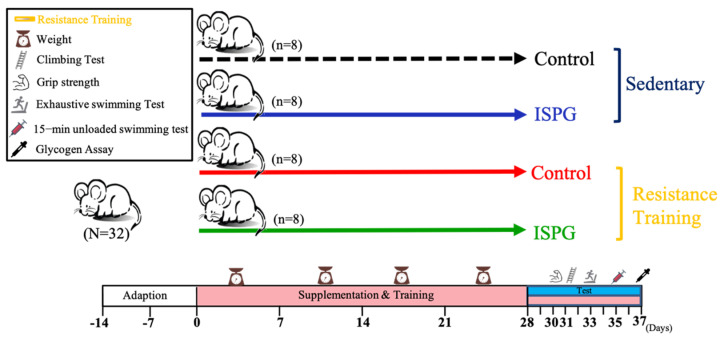
Experimental design.

The daily recommended amount of ISPG for a 60 kg human is 25 g, and the dose for mice is the recommended daily dose for humans based on the Human Equivalent Dose (HED) based on body surface area provided by the U.S. Food and Drug Administration. According to the guidance, the conversion coefficient between human and mice is 12.3 (available from https://www.fda.gov/media/72309/download, accessed date: 23 May 2019), thus the dosage for mice is day for 5.13 g/kg. All the mice were randomly divided into 4 groups (8 mice/group): (1) Sedentary control (SC), (2) ISPG, RT and ISPG + RT groups, respectively. All groups were accepted the same volume of distilled water or ISPG by oral gavage in 4 weeks (Figure 1). The amount of control and ISPG received by mice is same when calibrated by body weight.

### 2.3. Resistance Training Intervention and Test

We have modified the mouse standardization protocol in the RT and ISP + RT groups based on previous studies [8]. The mice were placed on the training equipment at angles (85 degrees) and about 100 cm in height. To avoid the use of ladders when weight-loading objects in the gaps of the ladder, we used a 1 cm^2^ square stainless steel, also within the length of the mouse step and also placed in 5 cm of water within the step length of the mouse to provide negative stimulation and increase motivation for climbing. The training objective consisted of muscle growth; namely, the adaptation and maximum muscle strength phases. The resistance training program as performed 5 days a week for 4 weeks and the indicated intensity load was adjusted through the spiral washers according to the weight of each animal, which was used to increase the load to adjust the intensity appropriately. The climbing procedures used 3 sets/day and 4 repetitions/set, with 1 min of rest between sets. In the first week of the beginning of the experiment for the adaptation phase, we subjected the mice to 5% BW weight-loading, then for the 2–4-week phase they were loaded with 25%, 50% and 75% BW, respectively. After 4 weeks of training, we used a similar approach to the training program, but did not load the weight to test the maximum number of climbs until fatigue and minimum climb time, to assess the anaerobic performance.

### 2.4. Forelimb Grip Strength

After all mice were treated with vector or ISPG for 28 days, forelimb grip strength of mice was measured by a low-force testing system (Model-RX-5, Aikoh Engineering, Nagoya, Japan) 30 min after feeding as in previous studies [9,10].

### 2.5. Exercise Endurance Test

After 4 weeks of supplementation with ISPG and RT, a lead sheet of 5% body weight was tied to the tail of each mouse, then was evaluated in a columnar swimming pool maintained at 28 ± 1 °C (65 cm high, 20 cm diameter, 40 cm deep). We recorded the time from entering the water to exhaustion for each mouse as endurance performance data. The mouse being not coordinated or unable to move in the water within 7 s was the criterion [11].

### 2.6. Determination of Fatigue-Associated Biochemical Variables

A 15-min swimming test was performed to evaluate biochemical biomarkers related to fatigue and tissue damage. Then, 30 min after the control or ISPG was administered, the mice completed 15 min of swimming. A blood sample was collected immediately after the swimming to obtain the serum which centrifuged for 15 min at 1500× *g*, 4 °C. The levels of serum lactate, ammonia, glucose (GLU), blood urine nitrogen (BUN), and activity of creatine kinase (CK) were determined by use of an auto-analyzer (Hitachi 717, Hitachi, Tokyo, Japan).

### 2.7. Body Composition, Glycogen Content and Histological Staining of Tissue

At the end of experiment, after all exercise tests were over (day 37 in Figure 1), all mice were treated for the last 30 min then were euthanized by 95% CO_2_, then the organs were accurately removed and weighed, including heart, lung, kidney, liver, MT (thigh muscle), muscle (gastrocnemius), BAT (brown adipocyte tissue) and EPF (epididymal fat pad). Organs were preserved in 10% formalin for standard fixation and dehydration, then embedded in paraffin, sliced at 4 µm thickness, and stained with hematoxylin and eosin (HE) for further histopathological identification. They were then examined by light microscopy with a charge-coupled device (CCD) camera (BX-51; Olympus, Tokyo, Japan) by a clinical pathologist. As in previous studies, part of the liver and muscle tissue was stored in liquid nitrogen for glycogen analysis [11].

### 2.8. Haematological Assessment

The mice were treated for the last time 30 min before being euthanized by 95% CO2 asphyxiation, and blood was collected by cardiac puncture after euthanasia. Blood was centrifuged for 15 min at 1500× *g*, 4 °C to obtain serum samples and analyze the level of aspartate amino transferase (AST), alanine aminotransferase (ALT), lactate dehydrogenase (LDH), CK, BUN, creatinine (CREA), uric acid (UA), total cholesterol (TC), triglyceride (TG), and GLU, and were measured by an auto-analyzer (Hitachi 717, Hitachi, Tokyo, Japan).

### 2.9. Statistical Analysis

Data are expressed as mean ± SEM and analyzed by two-way AVOVA with SPSS (version 23.0; SPSS, Chicago, IL, USA). A Tukey–Kramer test was used to measure the difference between different treatments. A Kruskal–Wallis test with a post-hoc Dunn’s test was used for multiple non-parametric comparisons. The statistical level was set at *p* < 0.05.

## 3. Results

### 3.1. Body Weight Dietary and Water Record

**Figure 2:**
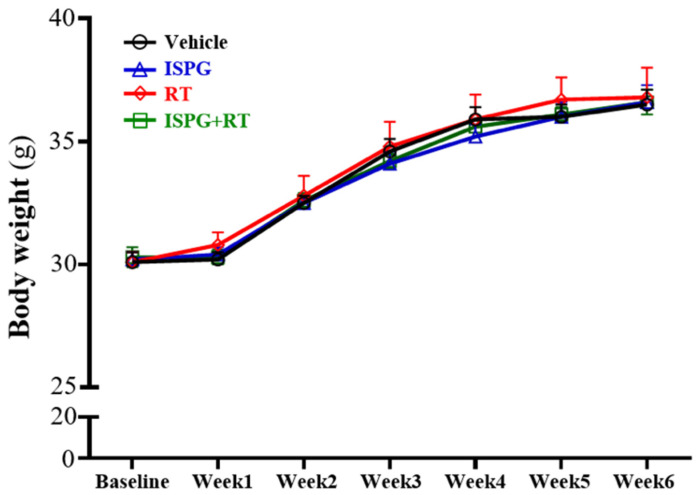
The effect of ISPG and resistance training on growth curve. Data were mean ± SEM for *n* = 8 mice per group.

From the beginning of the intervention to the end of the experiment, the weight of each group increased steadily, and there was no significant difference between the groups (Figure 2). In Table 2, The diet intake was of no significant difference between each group; however, the water intake in the ISPG group was significantly lower than in the ISPG + RT group (*p* = 0.0020), and those were all not significantly different from the SC group. The main effect of RT was significantly increased water intake and the main effect of ISPG was significantly decreased diet intake.

### 3.2. Effect of ISPG and RT on Grip Strength

**Figure 3:**
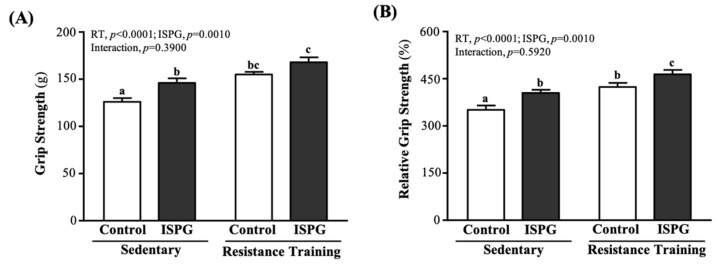
Effect of ISPG and resistance training on (**A**) absolute forelimb grip strength and (**B**) relative forelimb grip strength (%). All data are expressed as the mean ± SEM of 8 mice/group. Different superscript letters (a, b, c) above bars are significantly different at *p* < 0.05. If any letter is the same, it means that the difference between groups is not significant.

The mean forelimb grip strength in the SC, ISPG, RT and ISPG + RT groups was 126 ± 4, 146 ± 5, 155 ± 3 and 168 ± 5 g, respectively. Compared with SC, ISPG, RT and ISPG + RT groups were significantly higher by 1.16-fold (*p* = 0.0150), 1.23-fold (*p* < 0.0001) and 1.33-fold (*p* < 0.0001) (Figure 3A). The relative grip strength calculated by normalizing the weight of individual mice was also significantly higher with ISPG or RT (Figure 3B). The effects of ISPG and RT were significantly increased grip strength and relative grip strength (*p* < 0.05), but there was no interaction effect.

### 3.3. Effect of ISPG and RT on Swimming Endurance Test

**Figure 4:**
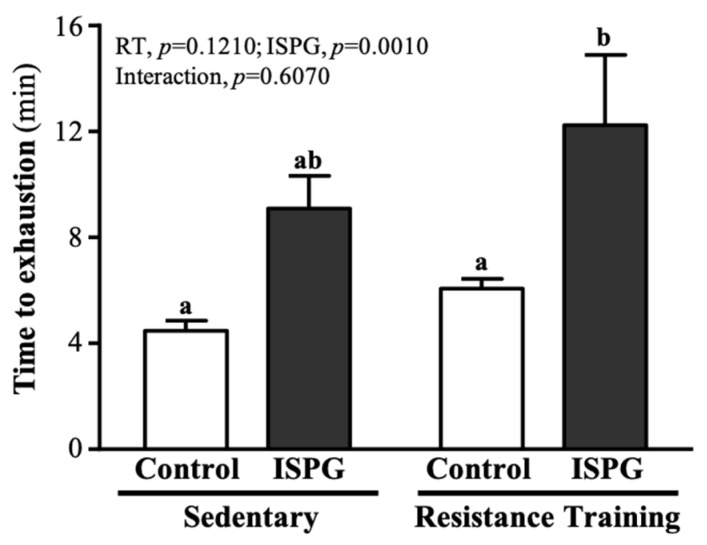
Effect of ISPG and resistance training on exhaustive swimming time. Data are expressed as the mean ± SEM of 8 mice/group. Different superscript letters (a, b) above the bar are significantly different at *p* < 0.05. If any letter is the same, it means that the difference between groups is not significant.

As seen in Figure 4, the endurance swimming time in the SC, ISPG, RT and ISPG + RT groups was 4.47 ± 0.39, 9.09 ± 1.24, 6.07 ± 0.37 and 12.24 ± 2.65 min, respectively. The swimming time of the ISPG + RT group was significantly greater than the SC group by 2.03-fold (*p* = 0.0050), but there was no significant difference between the ISPG, RT and SC groups. Only the ISPG effect could improve endurance exercise capacity (*p* = 0.0010).

### 3.4. Effect of ISPG and RT on Muscle Strength and Muscular Endurance Test

**Figure 5:**
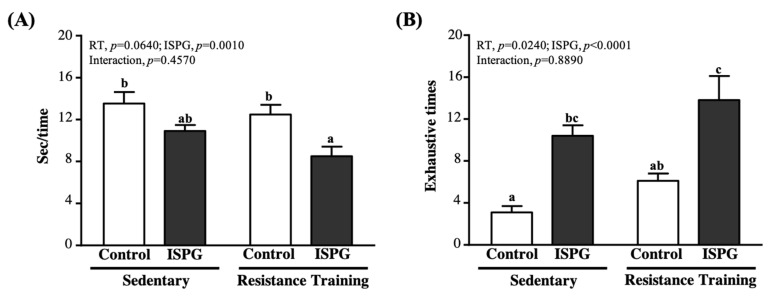
Effect of ISPG and resistance training on (**A**) muscle strength and (**B**) muscular endurance test. Data are expressed as the mean ± SEM of 8 mice/group. Different superscript letters (a, b, c) above the bar are significantly different at *p* < 0.05. If any letter is the same, it means that the difference between groups is not significant.

Anaerobic endurance and speed are used to assess muscle endurance performance and muscle strength. In terms of muscle strength performance, we took the speed of climbing as a consideration. As seen in Figure 5A, the average finished climbing times of SC, ISPG + RT groups were 13.52 ± 1.10, 10.91 ± 0.57, 12.47 ± 0.93 and 8.50 ± 0.90 s, respectively. Only the ISPG + RT group was significantly shorter than the SC group by 37.15% (*p* = 0.0001). As another index of muscular endurance performance (Figure 5B), we used the average maximum amount of climbing as representative. The repetition maximum (RM) of SC, ISPG, RT and ISPG + RT groups was 3.1 ± 0.6, 10.4 ± 1.0, 6.1 ± 0.7 and 13.8 ± 2.3 times, respectively. ISPG and ISPG + RT groups were not different but significantly greater than the SC group by 3.32-fold (*p* = 0.0030) and 4.40-fold (*p* = 0.0001). The effect of ISPG could promote muscle strength performance (*p* = 0.0010) and muscular endurance (*p* < 0.0001), and RT could promote muscular endurance (*p* = 0.0240).

### 3.5. Effect of ISPG and RT on Fatigue Indexes by 15 Min Exercise Challenge

**Figure 6:**
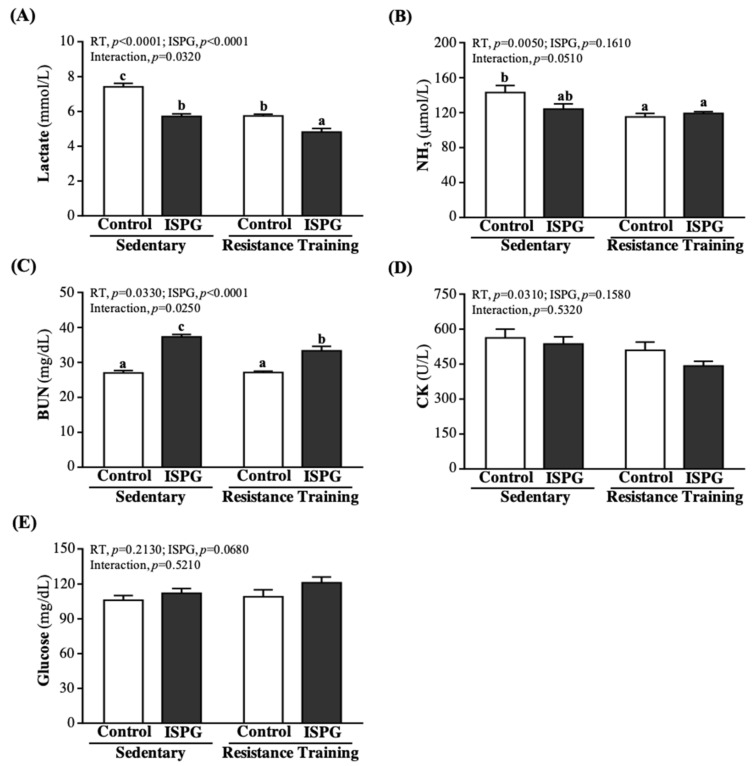
Effect of ISPG and resistance training on fatigue-related biochemical assessments of serum after 15-min acute exercise. (**A**) Lactate, (**B**) NH_3_, (**C**) BUN, (**D**) CK and (**E**) glucose. Data are expressed as the mean ± SEM of 8 mice/group. Different superscript letters (a, b, c) above the bar are significantly different at *p* < 0.05. If any letter is the same, it means that the difference between groups is not significant.

The evaluation results of fatigue-related indicators, including lactate, NH3, CK, BUN, and GLU, were performed immediately after 15 min of acute exercise. Serum lactate level was significantly lower by 22.90%, 22.62%, and 35.13% in the ISPG, RT, and ISPG + RT groups than the SC group (*p* < 0.0001) (Figure 6A). The NH_3_ level is shown in Figure 6B, RT and ISPG + RT groups were significantly lower than the SC group by 19.26% (*p* = 0.0070) and 16.99% (*p* = 0.0190). The BUN level was shown in Figure 6C, ISPG supplement groups were significantly higher than non-ISPG supplement groups, but combined with RT would decrease BUN level. In terms of glucose levels, ISP and ISPG +RT groups were significantly increased compared to the SC group by 1.38-fold (*p* < 0.0001) and 1.23-fold (*p* < 0.0001). As shown in Figure 6D, there was no significant difference between each group. The main effect of ISPG supplementation could significantly reduce lactate level (*p* < 0.0001) but increase BUN level (*p* < 0.0001). The RT could significantly reduce lactate (*p* < 0.0001), ammonia (*p* = 0.0050), CK (*p* = 0.0310), and BUN (*p* = 0.0330) level via the main effect of RT. The lactate (*p* = 0.0320) and BUN (*p* = 0.0250) had an interaction effect.

### 3.6. Effects of ISPG and RT on Body Composition

The organ weight was the basis for the body composition after sacrifice, including liver, kidney, heart, lung, gastrocnemius muscle, quadriceps, EFP and BAT. As shown on Table 2, there was no significant difference in liver, kidney, heart, lung, gastrocnemius muscle and BAT. After a 4-week resistance training, the quadriceps weight of SC, ISPG, RT, and ISPG + RT groups were 0.48 ± 0.06, 0.53 ± 0.04, 0.54 ± 0.05, and 0.55 ± 0.04 g, respectively. Compared with SC, only ISP + RT groups were significantly increased by 1.16-fold (*p* = 0.0140). The EFP of SC, ISP, RT, and ISP + RT groups were 1.37 ± 0.09, 0.89 ± 0.012, 0.83 ± 0.08, and 0.89 ± 0.07 g, respectively. ISPG, RT, and ISPG + RT groups were significantly reduced compared to SC by 33.58% (*p* = 0.0200), 38.97% (*p* = 0.0060), and 34.80% (*p* = 0.0150), with similar results after standardization by body weight. The main effect of ISPG was increased quadriceps weight (*p* = 0.0360) and percentage (*p* = 0.0130), and also decreased relative EFP weight (*p* = 0.0130). The main effect of RT was increased quadriceps weight (*p* = 0.0220) and percentage (*p* = 0.0150), and also decreased EFP (*p* = 0.0073). The interaction effect was significant on EFP of weight (*p* = 0.0190) and percentage (*p* = 0.0070).

### 3.7. Effects of ISPG and RT on Liver and Muscular Storage

**Figure 7:**
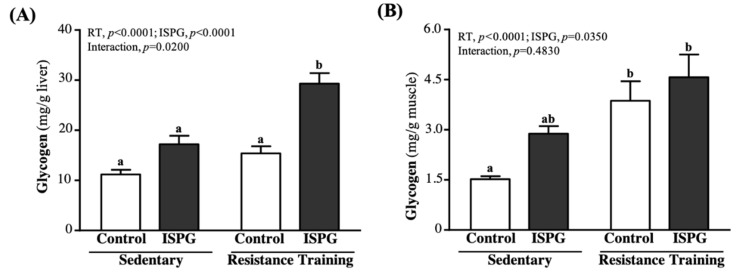
Effect of ISPG and resistance training on (**A**) hepatic and (**B**) muscle glycogen level. Data are expressed as the mean ± SEM of 8 mice/group. Different superscript letters (a, b) above the bar are significantly different at *p* < 0.05. If any letter is the same, it means that the difference between groups is not significant.

Glycogen is mainly found in liver and skeletal muscle tissue and is used for energy demand and homeostasis. The concentration of liver glycogen in the SC, ISPG, RT and ISPG + RT groups was 11.2 ± 0.9, 17.2 ± 1.7, 15.4 ± 1.4, 29.3 ± 2.1 mg/g, respectively (Figure 7A). The ISPG + RT group was significantly greater than the SC, ISPG, and RT groups by 2.61-fold (*p* < 0.0001), 1.70-fold (*p* < 0.0001) and 1.90-fold (*p* < 0.0001). In terms of muscle glycogen, as seen in Figure 7B, SC, ISPG, RT and ISPG + RT groups were 1.52 ± 0.09, 2.88 ± 0.23, 3.87 ± 0.58, 4.57 ± 0.68 mg/g, respectively. Compared with SC, RT and ISPG + RT groups were significantly greater by 2.54-fold (*p* = 0.0060) and 3.00-fold (*p* < 0.0001). The effect of ISPG and RT could significantly increase liver and muscle glycogen (*p* < 0.05), only liver glycogen had a significant interaction effect (*p* = 0.0200).

### 3.8. Effects of ISPG and RT on Clinical Biochemistries

At the end of the experiment, the serum was further analyzed to related clinical biochemistries, as shown in Table 3. The main and interaction effects of AST, ALT, TC, TG, CK, creatinine, UA, ALB, TP and GLU were not significantly different, and there was no significant difference between the groups shown. BUN level showed significant differences in ISPG main effect (*p* < 0.0001), with this effect significantly higher in the ISPG and ISPG + RT groups than the SC and RT groups. In terms of LDH index, the main effect significantly decreased in the RT (*p* = 0.0488) and ISPG + RT (*p* = 0.0256) groups than the ISPG group.

### 3.9. Effects of ISPG and RT on Histological Observation

**Figure 8:**
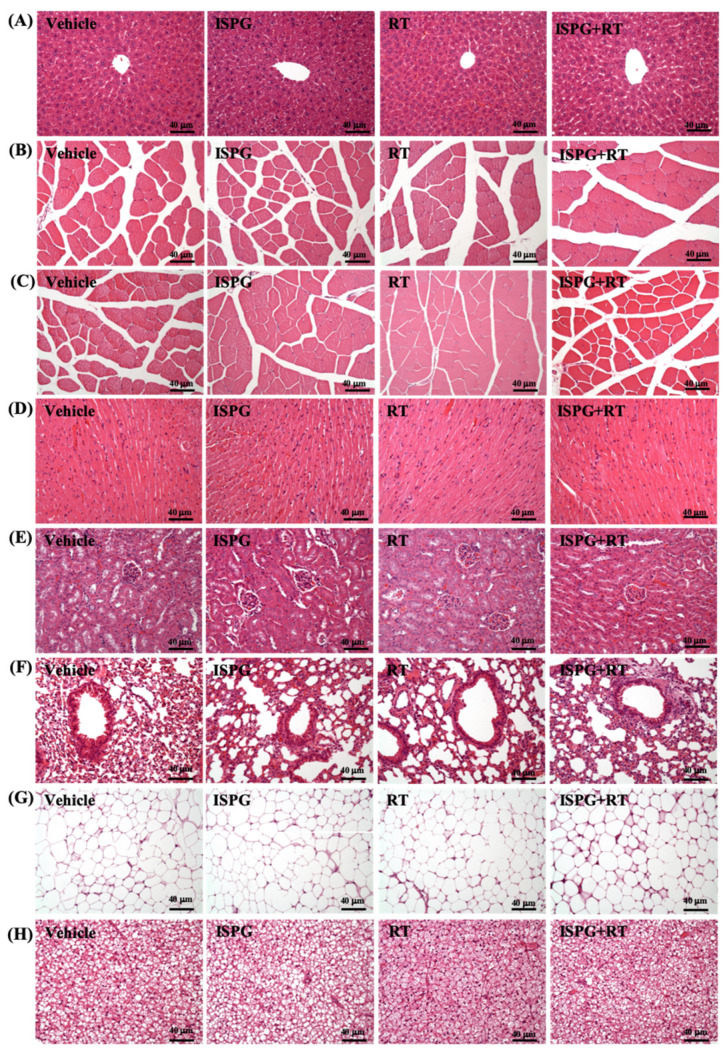
Effect of ISPG and RT on histomorphologic features of the (**A**) liver, (**B**) gastrocnemius muscle, (**C**) quadriceps, (**D**) heart, € kidney, (**F**) lung, (**G**) epididymal fay pads, and (**H**) brown adipose tissue. Specimens were photographed under a light microscope (HE stain, magnification: 200×; bar, 40 μm).

The toxicity of ISPG was evaluated by the histological staining of liver, kidney, heart, lung, brown adipose tissue, and epididymal fat pads (Figure 8). All the tissues did not differ among all groups.

## 4. Discussion

Past studies have shown that weight gain is not directly related to muscle mass and strength [12]. Therefore, we directly measured the weight of the EFP and muscle to determine the body composition of the mouse. At the end of the experiment, the body weight of each group increased steadily, but there was no difference between the groups (Figure 2). The EFP is a tissue in which male mice tend to deposit fat. We observed a significant difference in the weight of the four groups of EFP (Table 2). No matter ISPG supplementation or RT, the EFP in the ISPG, RT and ISPG + RT groups were significantly more reduced than the SC group. In addition, through resistance training and ISPG supplementation, we also found that the muscle mass of the quadriceps muscle could be increased without any difference in body weight. As many previous studies have shown, protein is built up by different types of essential amino acids and non-essential amino acids; among them, BCAA is important in mTOR activation to stimulate muscle synthesis, especially leucine [13]. Previous study has found that leucine could stimulate the muscle protein synthesis (MPS) efficiently [14]. The MPS rate is influenced by nutrition and exercise, especially the protein in the nutrition part [15]. The MPS rate is higher than the muscle protein breakdown (PMB) rate in the 3 h after the exercise training, the soy protein intake within the 3 h induced a much greater muscle anabolic response [16]. Proper resistance exercise training by supplementation with proteins can help improve muscle strength and quality [17,18]. The mechanism underpinning the effect might be the post-exercise ISPG administration promoting the MPS rate and muscle restoration from the exercise, which due to the energy depletion, caused muscle tissue damage and free radical accumulation. On the other hand, the ISPG promotes satiety to allow the mice to take less food which contains about 58% carbohydrate and 14% fat. However, the amount of food intake decreased in the ISPG-treated group, but the body weight of the growing mice did not decrease (Table 2). We think it might be that there was not much difference in the average daily total calorie intake between the groups. In the standard feed Chow 5001 we provided, each gram contains 3.36 kcal, and the difference in food intake was about 0.5–0.7 g, which was equivalent to 1.68–2.35 kcal. Calculated with the daily intake of 5.13 g/kg BW mice/day of ISPG in 30 g of mice, it was found that the average daily intake of ISPG calories in mice was 0.60 calories. Therefore, the average daily calorie intake of each mouse only differs by about 1.08–1.75 kcal. In addition, this study was only conducted for four weeks. Under the little calorie difference and short-term test, it should not easily cause a difference that results in body weight. Whether it would have an impact on long-term intervention may require further research and discussion.

The Asian Working Group for Sarcopenia (AWGS) noted that there is a positive correlation between muscle mass increase and grip strength performance improvement [19]. Our data showed that ISPG supplementation combined with RT could improve absolute and relative grip strength, and among them the ISPG + RT group had the highest forelimb grip strength than all groups (Figure 3), which represented the post-exercise ISPG administration to improve the forelimb grip strength. The benefits of ISPG and RT were not only reflected in muscle mass and strength, but also improved muscle explosiveness and muscular endurance (Figure 5). Previous study has pointed out that muscular power comes with endurance, explosiveness comes with power, speed comes with explosiveness and performance comes with speed [20]. The ISPG supplementation might have the benefit of exercise performance improvement. Especially associated with explosiveness, the ISPG + RT group finishing was the least time and maximum number of times. We could only speculate on the exact benefit of endurance and explosiveness improvement in combinations of exercise, but we need more research to clarify the intact relationship between exercise performance and the ISPG administration.

It is well known that sustained exercise stimulates several metabolisms in amino acid and protein [21]. The activation of branched-chain alpha-keto acid dehydrogenase (BCKDH) during exercise increased BCAA oxidation rate. Meanwhile, the BCKDH activity increased with prolonged exercise to lower the plasma and cellular leucine level [22]. The ISPG is a BCAA-rich protein which enhances the rest and post-exercise mTOR signaling activity [23]. In addition, leucine in ISPG can regulate the glucose oxidation rate in skeletal muscle through the stimulation of the glucose-alanine cycle (Cahill cycle) [24]. In Figure 4, the main effect on ISPG could increase aerobic exercise endurance capacity. We suggested that post-exercise ISPG supplementation can improve exercise performance through the MPS rate and energy utilization rate enhancement. Furthermore, ISPG is a combination of isolated soy protein and green tea. Study in post-exercise green tea administration has shown the catechin in green tea increases fatty acid oxidation rate to reduce the utilization rate of glycogen [23]. As a result, the catechin facilitates the glycogen recovery from exercise to relieve the fatigue. Energy depletion during exercise leads to muscle fatigue [25]. The skeletal muscle relies on fatty acid or carbohydrate as fuel during exercise. Especially medium to high intensity or prolonged exercise (> 90 min), the glycogen storage is considered as the limiting factor of energy utilization rate. Therefore, the endogenous glycogen storage was reported as a key role in endurance sports [26]. The glycogen can be synthesized from either glucose or trioses [27]. Glycogen is the main fuel of muscle during exercise which can generate adenosine triphosphate (ATP) through glycogen catabolism process. ATP is the sole fuel for muscle fiber contraction, of which the generation rate depends on the muscle and liver glycogen level during exercise [28] and ADP level increased after a prolonged medium intensity exercise [29]. We found the ISPG + RT group had both the highest liver and muscle glycogen levels among all groups (Figure 7A,B). Our data suggest that the storage amount of glycogen is improved through exercise training combined with ISPG administration. Meanwhile, glycogen and glucose are the main fuel of muscle during exercise, post-exercise blood glucose was evaluated to estimate the energy utilization in exercise [30]. We found both the ISPG group and the ISPG + RT group had a higher level of blood glucose (Figure 6E), which might be caused by the glucose spare effect of pre-exercise ISPG supplementation. In sum, ISPG supplementation has a benefit of increasing glycogen storage capacity to improve exercise performance.

It is commonly accepted that muscle fatigue plays an important role in exercise performance fluctuation [31]. Our previous research on anti-fatigue reported that the levels of serum lactate, ammonia, glucose, blood urea nitrogen (BUN) and creatine kinase activity are the biomarkers of fatigue [32]. Lactate is the main gluconeogenesis precursor when performing an anaerobic exercise [33]. During intense exercise, glucose is broken down to pyruvates, part of pyruvate is reduced by lactate dehydrogenase (LDH) to lactate as a result of a shortage of oxygen. NAD^+^ was regenerated for glycolysis through the reduction. However, lactate is an acidic compound which may interfere in the normal function of a cell and thus produce feelings of tiredness [34]. We observed ISPG, RT, and ISPG + RT groups had lower serum lactate levels than the SC group (Figure 6A), which may represent that ISPG can relieve muscle fatigue through the lowering of serum lactate level. Muscle fatigue is defined by the decline in maximal force or power output due to the abnormal muscle contraction when exposed to a prolonged or strenuous exercise, which involves peripheral and central fatigue. In our previous research, we reported that the serum ammonia level increased during exercise which is related to the peripheral and central fatigue [35]. In the current study, we found the ISPG, RT, and ISPG + RT groups had a lower level of serum ammonia when compared to the SC group (Figure 6B). Although both exercise and ISPG administration lowered the serum ammonia to relief the peripheral and central fatigue, we did not find a combination benefit of lowering ammonia in the ISPG + RT group. Many studies reported that strenuous exercise cause muscle damage, and creatine kinase and myoglobin in the muscle are released into the blood, thus the level of serum creatine kinase and myoglobin are considered as the reliable biomarkers of muscle damage [36,37]. In our study, we found that the ISPG + RT group had the least level of CK, although not a significant difference. However, we still suggested that pre-exercise ISPG supplementation can reduce muscle damage levels from strenuous exercise. BUN level is another index of fatigue, which is a common test for clinical kidney function evaluation. Since BUN is the degradation product of protein, it can also be the marker of the level of protein catabolism [38]. We found both the ISPG group and ISPG + RT group had the highest level of BUN. We suggested that part of ISPG was broken down during exercise to produce more waste products, which is the urea nitrogen. In conclusion, the pre-exercise ISPG administration had the benefit of reducing the fatigue biochemical parameters.

According to the results of this research, supplementation with ISP combined with resistance training could effectively improve muscle strength, muscle endurance, exercise endurance performance, glycogen storage, and delayed exercise fatigue biochemical indexes. It can be used as a sports nutrition supplement for sports or athletes. In the future, it could be used as long-term supplementation or further demonstration in clinical trials and extended as a sports nutrition supplement. In addition to verifying the improvement of exercise performance and delayed fatigue, we will further explore ways to reduce muscle pain or accelerate muscle strength recovery and antioxidant benefits.

## 5. Conclusions

In summary, the results of this study indicate that 28 days of ISPG combined with RT improves the performance of the forelimb grip strength, muscle strength and muscular endurance test and exhaustive-swimming test. Further, the ISPG + RT lowered serum ammonia and lactate level after the 15-min swimming test. Meanwhile, the ISPG + RT increased the muscle mass. In contrast, we found the combination of ISPG combined with RT had better benefits of muscle hypertrophy, forelimb grip strength and exercise performance when compared to the ISPG or RT group. The toxicity of ISPG was evaluated as safety in relevant parameters. Consequently, the ISPG could help promote muscle mass for the exerciser. In striving for muscle hypertrophy and improving exercise performance, the ISPG administration combined with exercise is a novel approach worth considering.

## Figures and Tables

**Table 1:** Nutrients, hydrolyzed catechin and isoflavones of ISPG.

Nutrients	Content/100 g ISPG
Protein	60.4 g
Fat	2.0 g
Total amount of saturated fatty acids	0.5 g
Carbohydrate	33.5 g
Total dietary fiber	3.0 g
Sodium	545 mg
Total calories	383.4 kcal
BCAA	g/100 g ISPG
Leucine	2.7
Isoleucine	2.9
Valine	3.0
0.46 g total catechin/100 g ISPG	mg/100 g ISPG
Catechin Gallate (CG)	-
Catechin (CT)	-
Epicatechin gallate (ECG)	1.79
Epicatechin (EC)	1.47
Epigallocatechin gallate	21.39
Epigallocatechin (EGC)	16.70
Gallocatechin (GCG)	-
Gallocatechin (GC)	1.01
Isoflavones	mg/100 g ISPG
Daidzin	3.75
Daidzein	5.00
Genistin	11.25
Genistein	10.00

**Table 2:** General characteristics of the experimental groups.

					*p* Values for Two-Way ANOVA		
Characteristic	Control +Sed	ISPG +Sed	Control +RT	ISPG +RT	Main Effect of RT	Main Effect of ISPG	Interaction (RT × ISPG)
**Initial BW (g)**	30.14 ± 0.38	30.24 ± 0.27	30.11 ± 0.42	30.29 ± 0.42	0.9740	0.7180	0.9220
**Final BW (g)**	37.28 ± 0.41	37.26 ± 0.81	37.65 ± 1.15	37.34 ± 0.25	0.7650	0.8290	0.8420
**Food intake (g)**	7.49 ± 0.31	6.95 ± 0.23	7.81 ± 0.30	6.99 ± 0.28	0.5220	0.0160 *	0.6150
**Water intake (mL)**	7.72 ± 0.09 ^ab^	6.95 ± 0.05 ^a^	7.76 ± 0.08 ^ab^	7.91 ± 0.10 ^b^	0.0050*	0.6180	0.0250 *
**Tissue weight (g)**							
**Liver**	2.06 ± 0.05	2.07 ± 0.09	2.17 ± 0.06	2.07 ± 0.07	0.4250	0.5430	0.4660
**Gastrocnemius muscle**	0.39 ± 0.02	0.40 ± 0.01	0.40 ± 0.02	0.41 ± 0.01	0.4730	0.5260	0.8990
**Quadriceps**	0.48 ± 0.02 ^a^	0.53 ± 0.01 ^a^	0.54 ± 0.02 ^a^	0.55 ± 0.01 ^b^	0.0220 *	0.0360 *	0.2890
**Heart**	0.19 ± 0.01	0.19 ± 0.01	0.19 ± 0.01	0.19 ± 0.00	0.8440	0.5560	0.5560
**Lung**	0.23 ± 0.01	0.23 ± 0.01	0.24 ± 0.01	0.24 ± 0.01	0.5030	0.9110	0.5770
**Kidney**	0.75 ± 0.04	0.79 ± 0.03	0.78 ± 0.03	0.75 ± 0.03	0.8930	0.8620	0.2940
**EFP**	0.51 ± 0.04 ^b^	0.34 ± 0.05 ^a^	0.31 ± 0.03 ^a^	0.33 ± 0.03 ^a^	0.0130 *	0.0620	0.0190 *
**BAT**	0.11 ± 0.01	0.11 ± 0.01	0.11 ± 0.01	0.12 ± 0.01	0.4820	0.4820	0.4820
**Relative tissue weight (%)**							
**Liver**	5.53 ± 0.14	5.54 ± 0.18	5.78 ± 0.17	5.55 ± 0.17	0.4160	0.5150	0.4690
**Gastrocnemius muscle**	1.04 ± 0.04	1.06 ± 0.03	1.05 ± 0.03	1.09 ± 0.04	0.4880	0.4070	0.8350
**Quadriceps**	1.28 ± 0.06 ^a^	1.43 ± 0.02 ^b^	1.42 ± 0.03 ^b^	1.48 ± 0.04 ^b^	0.0150 *	0.0130 *	0.2780
**Heart**	0.50 ± 0.02	0.51 ± 0.02	0.52 ± 0.03	0.50 ± 0.01	0.9760	0.6140	0.5730
**Lung**	0.61 ± 0.04	0.63 ± 0.02	0.65 ± 0.03	0.63 ± 0.03	0.5770	0.9050	0.6610
**Kidney**	2.00 ± 0.07	2.11 ± 0.06	2.07 ± 0.08	2.01 ± 0.08	0.8230	0.7570	0.2350
**EFP**	1.37 ± 0.09 ^b^	0.89 ± 0.12 ^a^	0.83 ± 0.08 ^a^	0.89 ± 0.07 ^a^	0.0070 *	0.0370 *	0.0070 *
**BAT**	0.29 ± 0.01	0.29 ± 0.02	0.29 ± 0.02	0.31 ± 0.01	0.4820	0.4820	0.5260

All data are expressed as the mean ± SEM of 8 mice/group. Different letters (^a^, ^b^) in the same column of data are significantly different, *p* < 0.05. If any letter is the same, it means that the difference between groups is not significant. The main effect is analyzed by two-variance analysis, * *p* < 0.05. EFP: epididymal fat pad; BAT: brown adipose tissue.

**Table 3:** Effects of ISPG and resistance training on biochemical assessments of serum at the end of the experiment.

					*p* Values for Two-Way ANOVA		
Parameter	Control +Sed	ISPG +Sed	Control +RT	ISPG +RT	Main Effect of RT	Main Effect of ISPG	Interaction (RT×ISPG)
**AST (U/L)**	66 ± 4	68 ± 2	67 ± 3	67 ± 5	0.9210	0.7670	0.8690
**ALT (U/L)**	56 ± 3	55 ± 3	53.5 ± 3	52 ± 3	0.1980	0.5280	0.8660
**TC (mg/dL)**	132 ± 3	130 ± 3	132 ± 5	128 ± 6	0.8950	0.4720	0.8250
**TG (mg/dL)**	130 ± 4	128 ± 4	127 ± 4	117 ± 4	0.0900	0.1770	0.3190
**CK (U/L)**	80 ± 5	81 ± 7	82 ± 4	83 ± 5	0.7020	0.8110	0.9620
**LDH (U/L)**	427 ± 37	511 ± 45	390 ± 41	373 ± 43	0.0440 *	0.4270	0.2299
**BUN (mg/dL)**	27.5 ± 0.6 ^a^	33.8 ± 1.5 ^b^	27.7 ± 0.8 ^a^	31.9 ± 0.9 ^b^	0.3980	<0.0001 *	0.2790
**CREA (mg/dL)**	0.45 ± 0.01	0.45 ± 0.01	0.43 ± 0.01	0.44 ± 0.01	0.1350	0.8420	0.6430
**UA (mg/dL)**	4.0 ± 0.2	4.2 ± 0.2	4.0 ± 0.3	4.1 ± 0.5	0.8300	0.6680	0.8580
**ALB (g/dL)**	3.1 ± 0.0	3.1 ± 0.0	3.1 ± 0.1	3.1 ± 0.0	0.6870	0.3510	0.8930
**TP (g/dL)**	5.1 ± 0.1	5.2 ± 0.1	5.1 ± 0.1	5.2 ± 0.1	1.0000	0.1430	0.8030
**GLU**	235 ± 12	249 ± 12	243 ± 17	286 ± 28	0.2210	0.1370	0.4280

All data are expressed as the mean ± SEM of 8 mice/group. Data are the same row with different letters (^a^, ^b^) differ significantly at *p* < 0.05 by two-way ANOVA. If any letter is the same, it means that the difference between groups is not significant. The main effect is analyzed by two-variance analysis, * *p* <0.05. AST, aspartate aminotransferase; ALT, alanine aminotransferase; TG, triacylglycerol; TC, total cholesterol; CK, creatine kinase; ALB, LDH, lactate dehydrogenase; albumin; BUN, blood urea nitrogen; CREA, creatine; UA, uric acid; TP, total protein; GLU, glucose.
